# ONC201 activates ER stress to inhibit the growth of triple-negative breast cancer cells

**DOI:** 10.18632/oncotarget.15451

**Published:** 2017-02-17

**Authors:** Xun Yuan, Dhonghyo Kho, Jing Xu, Ambikai Gajan, Kongming Wu, Gen Sheng Wu

**Affiliations:** ^1^ Department of Oncology, Tongji Hospital of Tongji Medical College, Huazhong University of Science and Technology, Wuhan, 430030, P.R. China; ^2^ Departments of Oncology and Pathology, Karmanos Cancer Institute, Wayne State University School of Medicine, Detroit, Michigan, 48201, USA

**Keywords:** ONC201, triple-negative breast cancer, TRAIL, ATF4, apoptosis

## Abstract

ONC201 was previously identified as a first-in-class antitumor agent and small-molecule inducer of the TRAIL (tumor necrosis factor-related apoptosis-inducing ligand) gene that induces apoptosis in cancer cells. ONC201 has a safety profile and is currently in phase II clinical trials for the treatment of various malignancies. In the current study, we examine the effect of ONC201 on triple-negative breast cancer cells (TNBC), a subtype of breast cancer that is sensitive to TRAIL. We find that ONC201 inhibits the growth of TNBC cells including TNBC cells that have developed acquired TRAIL resistance. However, TNBC cells that have developed acquired ONC201 resistance are cross-resistant to TRAIL. Mechanistically, ONC201 triggers an integrated stress response (ISR) involving the activation of the transcription factor ATF4. Knockdown of ATF4 impairs ONC201-induced apoptosis of TNBC cells. Importantly, the activation of ATF4 is compromised in ONC201-resistant TNBC cells. Thus, our results indicate that ONC201 induces an ISR to cause TNBC cell death and suggest that TNBC patients may benefit from ONC201-based therapies.

## INTRODUCTION

An estimated 246,660 women will be diagnosed with invasive breast carcinoma, and ∼40,450 will die from this disease in the US in 2016, making breast cancer the most common cancer and the second-most common cause of cancer deaths among women [1]. TNBC makes up approximately 15-20% of all breast cancer cases and most commonly affects younger women and African-American women. Patients with TNBC generally have a poor prognosis and short-term survival. TNBC lacks expression of the estrogen receptor, progesterone receptor, and HER2 [2, 3]. Consequently, this aggressive disease does not respond to widely used targeted therapies, such as trastuzumab, or endocrine therapies, such as tamoxifen or aromatase inhibitors [4, 5]. Conventional chemotherapeutic agents, including taxanes and anthracyclines, are standard-of-care treatment for TNBC [2]. Women with TNBC initially respond to these chemotherapeutic agents, but relapse is inevitable, and oncologists have little else to offer other than other non–cross-reactive standard chemotherapy regimens [4]. Therefore, the challenge is to develop a more effective treatment regimen for TNBC patients.

TNBC can be classified into at least 6 subtypes, including the basal (epithelial)-like, mesenchymal-like, and luminal androgen receptor subtypes [6]. Most TNBCs are mesenchymal-like [3, 6, 7]. Despite the lack of defined clinical targets, most TNBC cells are highly sensitive to TRAIL [tumor necrosis factor (TNF)-related apoptosis-inducing ligand]-induced apoptosis [8]. This feature warrants developing TRAIL as a targeted therapy for mesenchymal-like TNBC.

TRAIL (or Apo2 ligand) is a member of the TNF family [9, 10]. It selectively induces apoptosis in transformed or tumor cells but not in normal cells, making it a promising agent for cancer therapy [9–11]. We and others cloned four membrane-bound receptors for TRAIL, including death receptor 4 (DR4) [12], DR5 [13–18], TRID [15, 19–21], and TRUNDD [22–24]. When TRAIL binds to DR4 or DR5, it triggers the formation of a death-inducing signaling complex by recruiting Fas-associated protein with death domain (FADD) and caspase-8 or -10, resulting in activation of caspase-8 or -10, which leads to apoptotic cell death. Crosstalk between the death receptor and the mitochondrial apoptotic pathways via caspase-8-mediated Bid cleavage amplifies the TRAIL apoptotic signal. The potency and safety of treatment with TRAIL has prompted clinical trials with the recombinant protein as a novel treatment for human cancer [25]. Although TRAIL actively kills tumor cells, recombinant TRAIL possesses drug properties that limit its efficacy, such as short serum half-life, instability, and the inability to cross the intact blood-brain barrier. Furthermore, some cancer cells, including some types of TNBC, are intrinsically resistant to TRAIL, while others are initially sensitive to TRAIL but later acquire resistance through various mechanisms, including overexpression of members of the anti-apoptotic Bcl-2 family [26–37]. Thus, another challenge is how to overcome this acquired resistance to TRAIL.

ONC201 (or TIC10) was identified as a first-in-class antitumor agent and small-molecule inducer of the TRAIL gene that has been shown to have preclinical efficacy in a variety of cancer cells, including TNBC cells [38]. Our initial study showed that ONC201 inhibits the Akt/ERK pathways that ultimately induces apoptosis through the TRAIL/DR5 pathway [38]. Recent studies found that ONC201 can induce the ATF4-mediated integrated stress response to inhibit the growth of colorectal cancer cells and leukemia/lymphoma cells [39, 40]. ONC201 has several unique anticancer features, including a longer half-life, prolonged elevation of serum TRAIL, a bystander effect of TRAIL production by normal cells, and stimulation of TRAIL and DR5 expression [38]. ONC201 exhibits potent anticancer activity in a variety of cancer types in preclinical cancer models [38]. ONC201 was approved by the FDA for phase I/II clinical trials of treatments for several cancer types in 2014. Importantly, the phase I dose-escalation study was completed, and the safety of ONC201 was established. It is now in phase II trials in several types of cancer including breast cancer.

In this study we investigated the effects of ONC201 on TNBC cell growth and found that ONC201 effectively inhibits growth of TNBC cells. Importantly, TNBC cells that have acquired TRAIL resistance are still sensitive to ONC201. Furthermore, TNBC cells that have developed acquired ONC201 resistance are cross-resistant to TRAIL. The effects of ONC201 on TNBC cells were primarily due to its activation of ATF4-mediated ER stress response. Thus, our data suggest that ONC201 can be developed as a targeted agent for the treatment of TNBC.

## RESULTS

### Mesenchymal-like and epithelial-like TNBC cells are equally sensitive to ONC201

ONC201 was initially identified as a TRAIL-inducing compound [38] and mesenchymal-like TNBC cells are sensitive to TRAIL [8]. We determined whether ONC201 and TRAIL sensitivities are similar in a given TNBC cell line. We treated 10 TNBC cell lines with known TRAIL sensitivities with various doses of ONC201 for 3 days and cell proliferation was determined by MTT assays. As shown in Table 1, the IC50 values in 10 TNBC cell lines ranged from 0.032 to 10.12 μM, and 7 of 10 lines’ IC50 were under 5 μM, indicating that the majority of TNBC cells are sensitive to ONC201. Importantly, ONC201 sensitivities were not correlated with their corresponding TRAIL sensitivities. Moreover, ONC201 sensitivity was also not correlated with mesenchymal-like or epithelial-like status. For example, epithelial-like BT20 cells were sensitive to ONC201 at an IC50 of 0.263 μM although this cell line is resistant to TRAIL-induced apoptosis [8]. These results suggest that both mesenchymal-like and epithelial-like TNBC cells are susceptible to ONC201-induced death.

**Table 1:** ONC201 IC50 values in a panel of TNBC cells

Cell line	Subtype	TRAIL Sensitivity	ONC201 IC50 (μM)
SUM159	Mesenchymal	Sensitive	0.032
HCC70	Epithelial	Resistant	0.075
BT20	Epithelial	Resistant	0.263
HCC1806	Epithelial	Resistant	0.338
SUM149	Mesenchymal	Sensitive	0.391
HCC1937	Epithelial	Resistant	1.760
MDA157	Mesenchymal	Sensitive	2.340
MDA468	Epithelial	Resistant	6.270
MDA231	Mesenchymal	Sensitive	7.010
BT549	Mesenchymal	Sensitive	10.120

### ONC201 effectively inhibits the growth of MDA231 cells with acquired resistance to TRAIL

**Figure 1:**
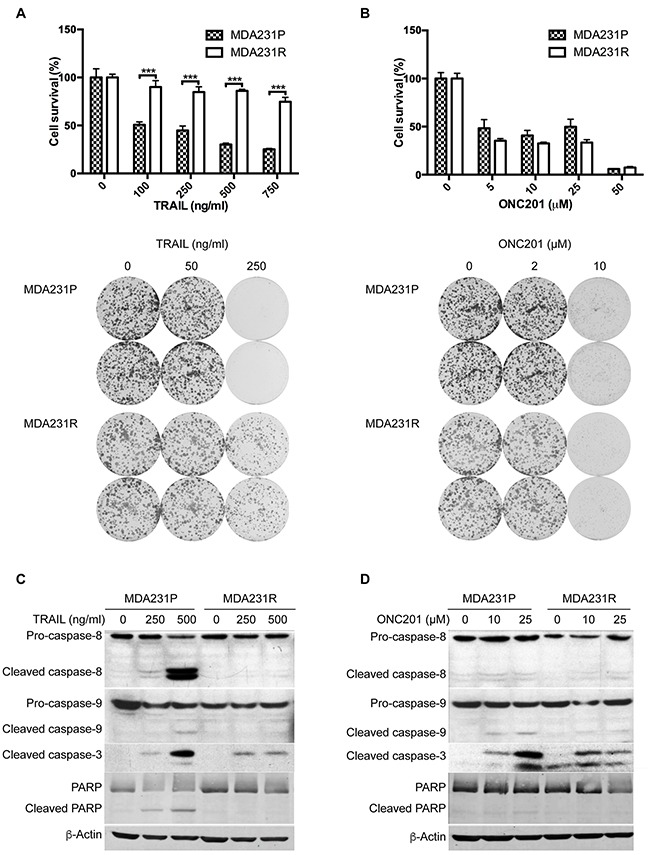
ONC201 induces apoptosis in TRAIL-sensitive and -resistant MDA231 cells MTT and colony formation analyses of MDA231P and MDA231R cells treated with TRAIL **A**. or ONC201 **B**. For MTT assays, MDA231P and MDA231R cells were left untreated or treated with 100, 250, 500, 750 ng/ml TRAIL or 5, 10, 25, 50 μM ONC201 for 72 h. For colony formation assays, 500 cells/well were seeded in 6-well plates. The next day, cells were left untreated or treated with 50, 250 ng/ml TRAIL or 2, 10 μM ONC201 for 72 h and then grown in medium without TRAIL or ONC201 for 12 days, followed by crystal violet staining. Data represent mean ± S.D. (*error bars*) of three independent experiments. ***, p<0.001, statistically significant. Western blot analyses of caspase-8, caspase-9, caspase-3 and PARP, treated with TRAIL **C**. or ONC201 **D**. MDA231P and MDA231R cells were left untreated or treated with 250 and 500 ng/ml TRAIL for 24 h or 10, and 25 μM ONC201 for 72 h. β-Actin was used as a loading control.

Our previous work indicated that ONC201 induces TRAIL/DR5 to activate TRAIL-mediated apoptosis [38, 41] and that TRAIL resistance including acquired resistance is a major cause of treatment failure [42]. To determine whether TNBC cells that have developed acquired resistance to TRAIL are also resistant to ONC201, we conditioned MDA231 cells to become resistant to TRAIL (MDA231R) by exposing the parental MDA231 cells (MDA231P) to gradually increasing concentrations of TRAIL, from 10 ng to 120 ng/ml, for over 6 months. As shown in Figure 1A, MDA231R cells did not respond to TRAIL-induced growth inhibition as compared to MDA231P cells, measured by MTT and colony formation assays. However, we found that ONC201 inhibited growth of both cell lines (Figure 1B). In fact, although MDA231R cells were resistant to TRAIL, they were even slightly more sensitive to ONC201 at doses of 5-25 μM, compared to MDA231P cells (Figure 1B). In addition, we found that ONC201 treatment activated caspase-3 and that the effects of ONC201 on caspase-3 activation were similar to TRAIL treatment in MDA231P cells (Figure 1C and 1D). However, the activation of caspase-8 by ONC201 was not significant as compared to MDA231P cells treated with TRAIL (Figure 1C and 1D), suggesting that caspase-8 may not play the major role in ONC201-induced apoptosis in TNBC cells. Given the fact that TRAIL resistance limits the successful development of TRAIL-based cancer therapies, our data suggest that ONC201 may be effective against TNBC cells that have acquired TRAIL resistance.

### ONC201-resistant TNBC cells are insensitive to TRAIL

**Figure 2:**
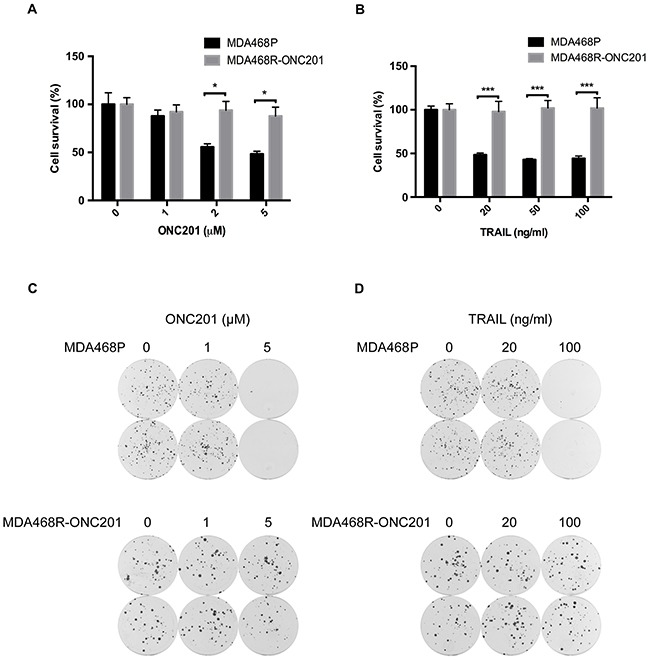
ONC201-resistant TNBC cells are insensitive to TRAIL MTT analyses of MDA468P and MDA468R-ONC201 cells treated with ONC201 **A**. or TRAIL **B**. Colony formation analyses of MDA468P and MDA468R-ONC201 cells treated with ONC201 **C**. or TRAIL **D**. For MTT assays, MDA468P and MDA468R-ONC201 cells were left untreated or treated with 20, 50, and 100 ng/ml TRAIL or 1, 2, and 5 μM ONC201 for 72 h. Data represent mean ± S.D. (*error bars*) of three independent experiments. *, P<0.05; ***, p<0.001, statistically significant. For colony formation assays, 500 cells/well were seeded in 6-well plates. After 24 h, cells were left untreated or treated with 20, 100 ng/ml TRAIL or 1, 5 μM ONC201 for 72 h, and then grown in medium without TRAIL or ONC201 for 12 days, followed by crystal violet staining.

Next, we asked if the development of ONC201 resistance affects TRAIL sensitivity. To this end, we established an ONC201-resistant MDA468 cell line (*i.e*., MDA468R-ONC201) by selection with increasing ONC201 doses over a 6-month period (Figure 2). We chose MDA468 cells because this cell line is sensitive to both TRAIL and ONC201. Compared to parental MDA468 cells (MDA468P), MDA468R-ONC201 cells are resistant to ONC201 (Figure 2A and 2C). Importantly, MDA468R-ONC201 cells were resistant to TRAIL-induced growth inhibition compared to MDA468P cells (Figure 2B and 2D). Similar results were obtained from MDA231 cells that we have generated for acquired ONC201 resistance (data not shown). These data suggest that once TNBC cells acquired ONC201 resistance, they no longer respond to TRAIL.

### ONC201 primarily inhibits the growth of TNBC cells through the activation of ATF4-mediated ER stress responses

**Figure 3:**
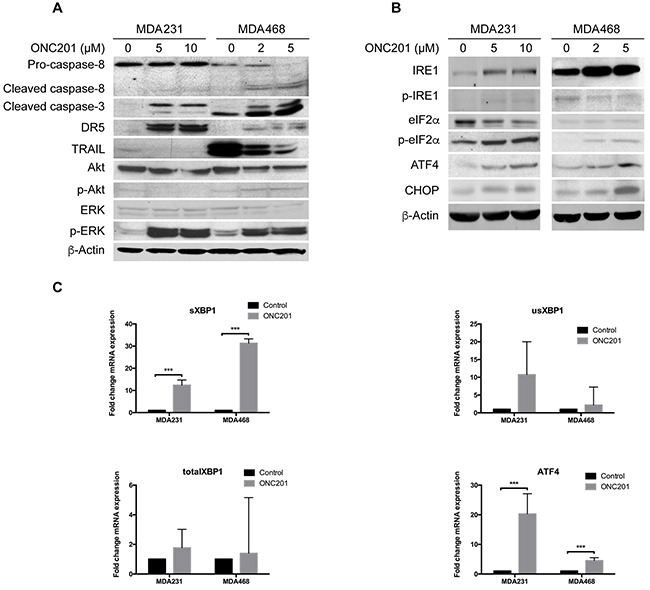
ONC201 activates ER stress pathways **A**. Western blot analyses of caspase-8, caspase-3, DR5, TRAIL, Akt, p-Akt, ERK and p-ERK. MDA231 and MDA468 cells were left untreated or treated with indicated concentrations of ONC201 for 72 h. β-Actin was used as a loading control. **B**. Western blot analyses of IRE1, p-IRE1, eIF2α, p-eIF2α, ATF4 and CHOP. MDA231 or MDA468 cells were left untreated or treated with ONC201 at indicated concentrations for 72 h. **C**, qRT-PCR analyses of the expression of *sXBP1*, *usXBP1*, total *XBP1* and *ATF4* in MDA231 and MDA468 cells treated with ONC201 (10 μM and 5 μM, 48 h) as compared with untreated cells. Fold changes were normalized relative to GAPDH.

To understand the mechanism by which ONC201 inhibits the growth of TNBC cells, we first examined the activation of the TRAIL apoptotic pathway because ONC201 was initially identified as a small-molecule inducer of TRAIL and DR5 by inactivating ERK and Akt to trigger TRAIL-induced apoptosis [38]. To this end, we treated MDA231 and MDA468 cells with ONC201, and the effects of ONC201 on the levels of these proteins were measured. Figure 3A shows that the levels of phosphorylated Akt (p-Akt) were inhibited in MDA231 cell but not in MDA468 cells by ONC201 treatment. Interestingly, ONC201 caused ERK phosphorylation/activation in both cell lines, and TRAIL levels were significantly decreased in ONC201-treated cells compared to untreated ones. In addition, DR5 was significantly induced by ONC201 in MDA231 cells but minimally in MDA468 cells. Furthermore, the effects of ONC201 treatment on the levels of caspase-8 cleavage were also not significant (Figure 3A), as compared to the cells treated with TRAIL in which caspase-8 was cleaved significantly (Figure 1C). However, caspase-3 cleavage was easily detected in ONC201-treated cells, indicating that apoptosis is induced by ONC201. Since the effects of ONC201 on the levels of Akt, ERK, TRAIL and DR5 are not consistent with its ability to inhibit the growth of TNBC cells, our data suggest that the activation of the AKT/ERK-TRAIL/DR5 axis may not be the major pathway by which ONC201 inhibits the growth of TNBC cells.

We then tested the effects of ONC201 on the activation of ER stress because two recent studies showed that ONC201 triggered an integrated stress response (ISR) to inhibit the growth of leukemia/lymphoma or colorectal cancer cells [39, 40]. Figure 3B shows that the levels of IRE1, p-IRE1, p-elF2a, ATF4 and CHOP proteins were induced by ONC201 to various degrees in both MDA231 and MDA468 cells, with IRE1 and ATF4 being significantly increased. An increase in ATF4 is consistent with recent studies in other cancer cell types including leukemia, lymphoma and colorectal cancer cells [39, 40]. Importantly, increased ATF4, XBP1 and spliced XBP1 (sXBP1) mRNAs by ONC201 were confirmed by real-time PCR (Figure 3C). Considering that p-elF2a/ATF4/CHOP axis and IRE1/XBP1/sXBP1 axis are two distinct ER stress pathways, our data suggest that ONC201 activates two ER stress pathways in TNBC cells.

**Figure 4:**
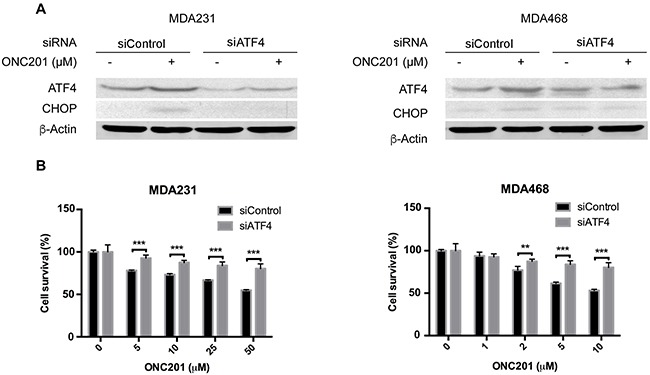
ATF4 knockdown decreases ONC201-induced growth inhibition **A**. Western blot analysis of ATF4 and CHOP in MDA231 and MDA468 cells transfected with siRNA against ATF4 or non-target siRNA, followed by treatment with ONC201 (10 μM, 24 h). **B**. MTT assays of MDA231 and MDA468 cells transfected with non-target siRNA or siRNA against ATF4 and then treated with ONC201 at the indicated concentrations for 72 h.

To further define the role of activation of ER stress in ONC201-induced cell death, we employed siRNA to assess the requirement of ER stress responses in ONC201-induced TNBC cell growth inhibition. Figure 4 shows that ATF4 was effectively knocked down in MDA231 cells by siRNA and that knockdown of ATF4 decreased ONC201-induced growth inhibition as compared to cells transfected with non-targeted siRNA under the same treatment condition. Similarly, siRNA-mediated knockdown of ATF4 in MDA468 while not pronounced led to significant reversal of the growth inhibition by ONC201. Since IRE1 was induced by ONC201 treatment, we knocked down IRE1 by siRNA and found that knockdown of IRE1 did not have an effect on ONC201-induced growth inhibition (Supplementary Figure 1), indicating that the activation of IRE1-mediated ER stress is not involved in ONC201-induced cell death. Thus, our data suggest that the p-elF2a/ATF4/CHOP axis, but not IRE1/XBP1/sXBP1 axis, likely plays a role in ONC201-induced growth inhibition in TNBC cells.

### Acquired ONC201-resistant TNBC cells have a defect in the activation of ATF4-mediated ER stress response

**Figure 5:**
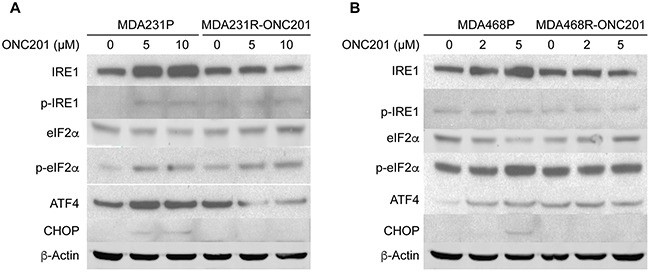
ONC201-resistant TNBC cells have a defect in the activation of ATF4 Western blot analyses of IRE1, p-IRE1, eIF2α, p-eIF2α, ATF4, and CHOP. MDA231P and MDA231R-ONC201 cells **A**. or MDA468P and MDA468R-ONC201 cells **B**. were left untreated or treated with the indicated concentrations of ONC201 for 72 h. β-Actin was used as a loading control.

To determine if the activation of the ER stress response plays a role in acquired ONC201 resistance, we examined the levels of ER stress responses in MDA231P and MDA231R-ONC201 cells. Consistent with the activation of ER stress (Figure 3B), the levels of IRE1, ATF4 and CHOP were increased in ONC201-treated parental MDA231 cells (MDA231P) (Figure 5A). In contrast, activation of ATF4 and IRE1 were compromised in ONC201-resistant MDA231 cells (MDA231R-ONC201), as exemplified by the levels of ATF4, CHOP and IRE1 (Figure 5A). Similar results were obtained with ONC201-resistant MDA468 cells as compared to parental MDA468 cells (Figure 5B). Taken together, these data suggest that ATF4-mediated ER stress response is compromised in cells that have developed acquired ONC201 resistance.

### ONC201-resistant TNBC cells are sensitive to conventional chemotherapeutics

**Figure 6:**
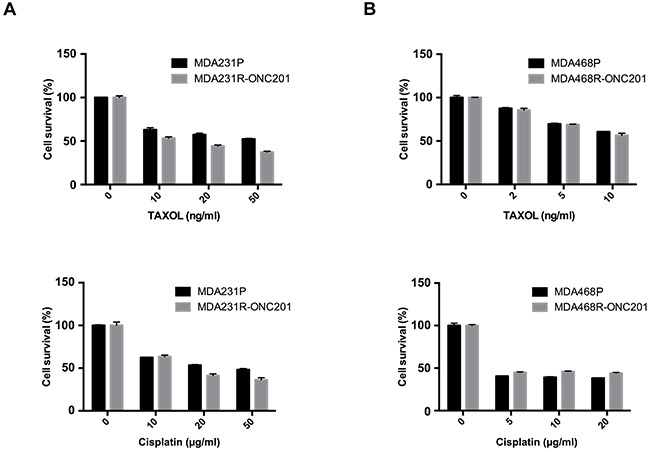
ONC201-resistant TNBC cells are sensitive to conventional chemotherapeutics MTT of parental and ONC201-resistant MDA231 **A**. and MDA468 **B**. cells treated with TAXOL or cisplatin. MDA231P, MDA231R-ONC201, MDA468P and MDA468R-ONC201 cells were left untreated or treated with the indicated concentrations of TAXOL or cisplatin for 72 h. Data represent mean ± S.D. (*error bars*) of three independent experiments.

To determine if ONC201-resistant TNBC cells respond to clinically used chemotherapeutics, we treated the parental and ONC201-resistant MDA231 and MDA468 cells with various doses of TAXOL and cisplatin for 3 days, and cell proliferation was then determined by MTT. As shown in Figure 6, the growth of both MDA231P and MDA231R-ONC201 were effectively inhibited by TAXOL or cisplatin. Similarly, both MDA468P and MDA468R-ONC201 showed comparable sensitivity. These results suggest that ONC201-resistant TNBC cells are susceptible to conventional chemotherapeutics.

## DISCUSSION

Previous studies have shown that TNBC cells are sensitive to TRAIL-induced apoptosis [8, 43]. However, it was not known if ONC201, a small-molecule inducer of the TRAIL gene, also induces apoptosis in TNBC cells. In this study, we used several TNBC cell lines to demonstrate that ONC201 effectively inhibits the growth of TNBC cells through the activation of ER stress response. Importantly, acquired TRAIL-resistant TNBC cells are still sensitive to ONC201. Thus, our data suggest that ONC201 is effective against TNBC.

Preclincal studies have established that the activation of the TRAIL pathway selectively induces apoptosis in cancer or transformed cells but not normal cells [42]. As a result, clinical trials using recombinant TRAIL or its agonistic antibodies against DR4 or DR5 as a single agent or in combination with clinically used chemotherapeutic agents were initiated in patients with different cancers [44, 45]. The safety of these TRAIL-based agents was established, but they had limited efficacy. It is noteworthly that all those clinical trials were conducted in all unselected patients [44, 45]. It is established that although TRAIL selectively kills cancer cells but not normal cells, most cancer cells are resistant to TRAIL. For example, breast cancer cells, in general, are resistant to TRAIL, but only TNBC cells are sensitive to TRAIL. In addition, TNBC cells have several subtypes, including the basal (epithelial)-like, mesenchymal-like, and luminal androgen receptor subtypes [3, 6, 7]. It has been shown that only mesenchymal-like TNBC cell are sensitive to TRAIL [8]. Thus, it is conceivable that if TRAIL-based clinical trials were conducted in patients with mesenchymal-like TNBC, those patients may likely benefit from the treatments.

It has been known that TNBC lacks defined therapeutic targets, but mesenchymal-like TNBC cells are highly sensitive to TRAIL. Hence, mesenchymal-like TNBC might be susceptible to TRAIL-targeted therapy [8]. We showed that ONC201 inhibited the growth of mesenchymal-like TNBC cells. We also showed that TRAIL-sensitive TNBC cells can develop acquired resistance after chronic exposure to TRAIL, but ONC201 still effectively inhibited the growth of TRAIL-resistant MDA231 cells. In addition, in spite of the fact that mesenchymal-like TNBC cells are sensitive to TRAIL, epithelial-like TNBC cells are resistant to TRAIL [8, 46]. However, we found that ONC201 inhibited the growth of MDA468 cells, suggesting that ONC201 can be effective against both mesenchymal-like and epithelial-like TNBC cells. Thus, we conclude that ONC201 has a broader antitumor activity than TRAIL against TNBC cells.

Mechanistically, ONC201 induces cell death via TRAIL-dependent and TRAIL-independent pathways [47]. In the TRAIL-dependent pathway, ONC201 inhibits Akt/ERK to activate FOXO3a-mediated transcriptional induction of TRAIL and subsequent TRAIL/DR5-mediated apoptosis [38]. In this study, we showed that ONC201 had a modest effect on the levels of phosphorylated Akt in MDA231 cells but not in MDA468 cells. However, we found that ONC201 increased rather than inhibited ERK phosphorylation in both cell lines. These data indicate that the main mechanism of ONC201-induced apoptosis is unlikely due to its ability to inhibit Akt/ERK to induce TRAIL expression. Consistent with this, we found that TRAIL levels were decreased rather than induced by ONC201. Interestingly, DR5 was induced by ONC201. ERK signaling has been demonstrated to positively regulate DR5 expression [48, 49]. ONC201 may induce DR5 through activation of ERK signaling. Thus, these data suggest that ONC201-induced anticancer activity may be in part through the TRAIL ligand-independent and DR5-dependent apoptotic pathway.

In the TRAIL-independent pathway, ONC201 induces ER stress responses via ATF4 to kill cancer cells [39, 40]. The ER stress response is one of the unfolded protein responses that mediate cell survival in the face of stressful stimuli [50]. ER stress is initiated by three ER transmembrane receptors—protein kinase RNA (PKR)-like ER kinase (PERK), activating transcription factor 6 (ATF6), and inositol-requiring enzyme 1 (IRE1). In resting cells, all three ER stress receptors bind to the ER chaperone GRP78 to maintain the cells in an inactive state. In response to ER stress, GRP78 dissociates from these three receptors to initiate ER stress responses through three distinct signaling pathways—the PERK-elF2-ATF4 pathway, the ATF6 pathway, and the IRE1-XBP1-sXBP1 pathway [51]. We showed that ONC201 activates ER stress responses, including IRE1-mediated ER stress. However, we found that knockdown of IRE1 did not affect ONC201 sensitivity in MDA231 cells, suggesting that IRE1 may not play a major role in mediating ONC201 antitumor activity. On the other hand, we showed that ATF4 and CHOP were induced by ONC201, and that knockdown of ATF4 attenuated ONC201-induced growth inhibition of MDA231 cells, indicating that ATF4-mediated ER stress is involved in ONC201-induced anticancer activity. These results are consistent with two recent studies in other cancer types showing that the activation of ATF4-mediated ER stress is required for ONC201-induced antitumor activity [39, 40]. Thus, we conclude that ONC201 activates two ER stress pathways, but the activation of ATF4-mediated ER stress is responsible for ONC201-induced antitumor activity in TNBC cells. Taken together, our data suggest that ONC201 activates TRAIL-dependent and TRAIL-independent pathways to inhibit the growth of TNBC cells.

A recent study indicated that TNBC, relative to other subtypes of breast cancer, exhibits an intrinsically higher level of ER stress response [52]. This unique activation pattern suggests that ER stress is a biomarker and could be a potential target for the treatment of TNBC. However, targeting ER stress as a novel therapeutic strategy for the treatment of TNBC has not been explored. Therefore, patients with TNBC might benefit from agents that can target the ER stress response. It is conceivable that ONC201, an antitumor agent and small-molecule inducer of the TRAIL gene, is such an “ideal” agent because it can induce ER stress responses while modulating the Akt/ERK signaling pathways. In addition, we showed that ONC201-resistant cells are still sensitive to clinically used chemotherapeutics, suggesting that ONC201 resistant tumors can be effectively inhibited by conventional chemotherapies.

In summary, this study makes the following novel observations: 1) ONC201 effectively inhibits the growth of TNBC cells including epithelial-like TNBC cells; 2) ONC201 inhibits the growth of TNBC cells that have developed acquired TRAIL resistance; 3) acquired ONC201 resistance in TNBC cells have a defect in the activation of ATF4-mediated ER stress; and 4) acquired ONC201 resistant TNBC cells are sensitive to conventional chemotherapy. Since TNBC cells have a higher level of ER stress than other subtypes of breast cancer, our study has an important implication. Thus, developing ER stress-inducing compounds, such as ONC201, can be novel strategies to treat TNBC. Importantly, ONC201 together with conventional chemotherapies suggests a novel treatment regimen for TNBC patients.

## MATERIALS AND METHODS

### Cell culture and reagents

Human breast cancer cell lines were obtained from the Biobank and Correlative Sciences Core, Wayne State Univeristy, Karmanos Cancer Institute. MDA231, MDA468 and MDA157 cells were cultured in DMEM supplemented with 10% fetal bovine serum (FBS). SUM159 and SUM149 cells were cultured in F12 supplemented with 5% FBS, 1mg/ml hydrocortisone and 5 mg/ml insulin. HCC70, HCC1937 and HCC1806 cells were maintained in RPMI-1640 supplemented with 10% FBS. BT549 cells were cultured in RPMI-1640 supplemented with 10% FBS and 0.85 mg/ml insulin. BT20 cells were maintained in MEM supplemented with 10% FBS. All cells were cultured in media supplemented with amphotericin B and gentamicin. ONC201 was obtained from Oncoceutics, Inc. (Philadelphia, PA). Recombinant human TRAIL/Apo2L was obtained from Peprotech, Inc. (Rocky Hill, NJ). Taxol was obtained from the Oncology Outpatient Pharmacy at Karmanos Cancer Institute. Cisplatin and β-Actin antibody was purchased from Sigma (St Louis, MO). Rabbit anti-caspase-8, caspase-9, caspase-3, DR5, Akt, p-Akt, ERK, p-ERK, IRE1, and eIF2α antibodies were obtained from Cell Signaling Technology (Danvers, MA). Rabbit anti-phospho-IRE1 antibody was from Thermo Fisher Scientific (Waltham, MA). Rabbit anti-p-eIF2α antibody was from Abcam (Cambridge, UK). Rabbit anti-CHOP and ATF4 antibodies and mouse anti-PARP antibody were obtained from Santa Cruz Biotechnology, Inc. (Santa Cruz, CA).

### Generation of TRAIL-resistant MDA231 cells, ONC201-resistant MDA231 cells and ONC201-resistant MDA468 cells

Parental MDA231 cells (MDA231P) were gradually exposed to increased concentrations of TRAIL starting from 10 to 120 ng/ml for over 6 months to select TRAIL-resistant cells (MDA231R). Likewise, ONC201-resistant MDA231R-ONC201 and MDA468R-ONC201 cells were established by chronically exposing parental MDA231 cells and MDA468 cells to gradually increased concentrations of ONC201 starting from 1 μM to 5 μM for over 6 months.

### RNA Interference of ATF4 and IRE1

On-TARGET plus SMART pool siRNAs for ATF4 and corresponding control non-target siRNA were purchased from Dharmacon Research (Lafayette, CO). Pooled siRNA for IRE1 and corresponding control non-target siRNA were purchased from Santa Cruz Biotechnology, Inc. (Santa Cruz, CA). Transfections were performed as suggested by the manufacturer's instruction with slight modifications as described previously (11). Briefly, cells were seeded in 6-well plates (5×10^5^ cells/well). The next day, cells were transfected with siRNA oligonucleotides using Lipofectamine RNAiMAX (Invitrogen) and transfected cells were left untreated or treated with ONC201. After 3 days, they were harvested for protein expression measurement by Western blot analysis. For ONC201 sensitivity experiments, transfected cells were placed at 5,000 cells/well in 96-well plates and then left untreated or treated with ONC201, and cell proliferation was determined by MTT assay.

### Cell viability assay and colony formation assay

MTT assay was performed as described previously (12). Briefly, cells were left untreated or treated with TRAIL or ONC201. After incubation with MTT solution for 4 h, isopropyl alcohol was added to dissolve the formazan crystals. OD was measured using a V_max_ Microplate Reader (Molecular Devices, Sunnyvale, CA) at 570 nm. The cell proliferation was calculated from the mean of pooled data from three separate experiments with four wells each. The colony formation assay was described previously (13). Briefly, cells were plated (5×10^2^ cells/well) in 6-well plates. The next day, cells were treated with vehicle, TRAIL, or ONC201. After 3 days of incubation with drugs, the medium was changed. After 14 days, colonies were stained with 0.25% crystal violet in 10% formalin and 80% methanol for 2 hr, washed, and counted. Colonies containing 50 cells or more were counted. The results represented at least three independent experiments.

### Western blot analysis

Cell lysates were prepared as previously described (12), and protein concentration was determined using the Protein Assay Kit (Bio-Rad, Hercule, CA). Cell lysates were electrophoresed through denaturing polyacrylamide gels and transferred to a PVDF membrane (Millipore, Bedford, MA). The blots were probed or re-probed with the antibodies, and detected using Enhanced Chemiluminescence (ECL) or Odyssey Infrared Imaging System according to the manufacturer's protocol.

### Isolation of RNA and real-time-PCR

Total cellular RNA was purified from cells using the RNeasy Mini Kit (Qiagen, Hilden, Germany). Template cDNA was synthesized from total RNA SuperScript™ III Reverse Transcriptase (Invitrogen, Carlsbad, CA) according to the manufacturer's instructions. Real-time PCR was performed using SYBR^®^ Green PCR Master Mix (Applied Biosystems) and specific primers directed against human sXBP1 (5′-CTGAGTCCGAATCAGGTGCAG-3′ and 5′-ATC CATGGGGAGATGTTCTGG-3), usXBP1 (5′-CAGCAC TCAGACTACGTGCA-3′ and 5′-ATCCATGGGGAGATG TTCTGG-3′), total XBP1 (5′-TGGCCGGGTCTGCT GAGTCCG-3′ and 5′-ATCCATGGGGAGATGTTCTG G-3′), and ATF4 (5′-TGGCCAAGCACTTCAAACCT-3′ and 5′-GTTGTTGGAGGGACTGACCAA-3′). Gene expression was normalized to GAPDH. All reverse transcription (RT) reactions, including no-template controls and RT minus controls, were run according to the standards specified by Applied Biosystems. Each sample was tested in triplicate and data were analyzed according to the ΔΔCt method.

### Statistical analysis

Statistical analysis was performed using Student's *t* test. The data were presented as the mean ± S.D., and *p* ≤ 0.05 was considered significant. All statistical analyses were performed with Graphpad Prism version 6.0c.

## SUPPLEMENTARY MATERIALS FIGURE



## Abbreviations

TRAIL: tumor necrosis factor-related apoptosis-inducing ligand
TNBC: triple-negative breast cancer
ISR: integrated stress response
FADD: Fas-associated protein with death domain
PERK: protein kinase RNA-like ER kinase
ATF4: activating transcription factor 4
IRE1: inositol-requiring enzyme 1
CHOP: C/EBP homologous protein.
