# Supplementary material for: ONC201 activates ER stress to inhibit the growth of triple-negative breast cancer cells
Source: Oncotarget. 2017 Feb 17;8(13):21626–38. doi: 10.18632/oncotarget.15451 (PMC5400611; doi:10.18632/oncotarget.15451)
Supplement: Supplementary file 1 [file oncotarget-08-21626-s001.pdf]

ONC201 activates ER stress to inhibit the growth of triple-negative breast cancer cells

SUPPLEMENTARY FIGURE

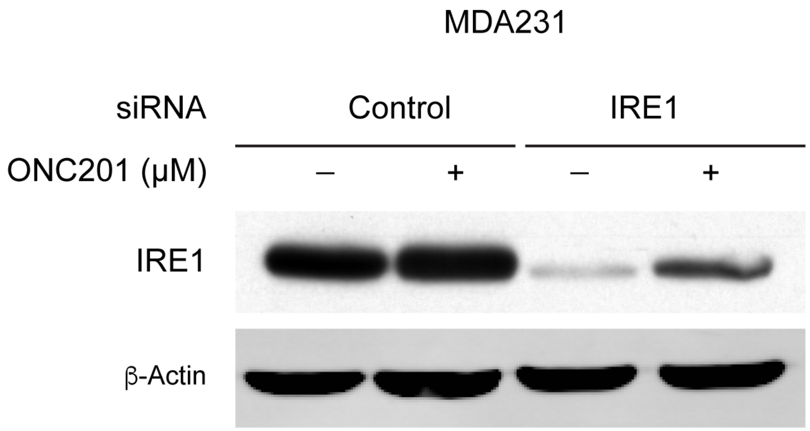

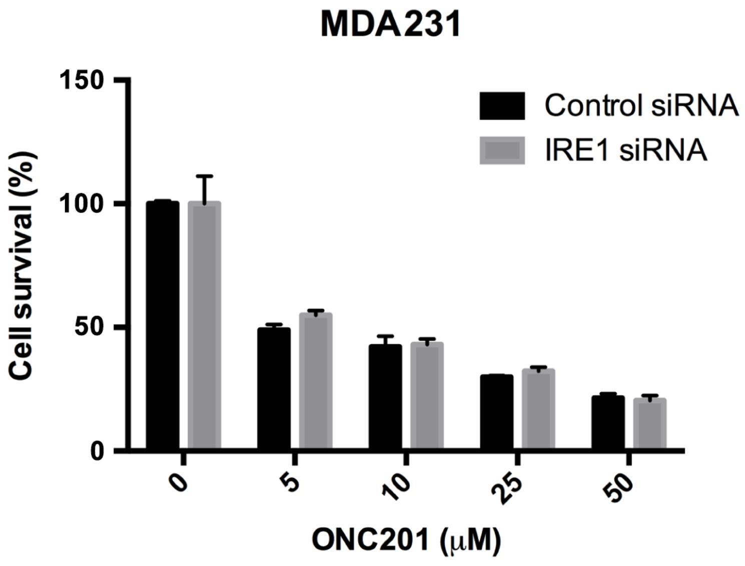

Supplementary Figure 1: IRE1 knockdown does not impact ONC201-induced growth inhibition. Upper Panel. Western blot analysis of IRE1 in MDA231 cells transfected with non-target siRNA or siRNA against IRE1, followed by treatment with ONC201(10μM, 48h). **Lower Panel.** MTT assay of MDA231 cells transfected with non-target siRNA or siRNA against IRE1 and then treated with ONC201 at the indicated concentrations for 72h.
